# Endothelial progenitor cells display clonal restriction in multiple myeloma

**DOI:** 10.1186/1471-2407-6-161

**Published:** 2006-06-22

**Authors:** Marc Braunstein, Tayfun Özçelik, Sevgi Bağişlar, Varsha Vakil, Eric LP Smith, Kezhi Dai, Cemaliye B Akyerli, Olcay A Batuman

**Affiliations:** 1 Division of Hematology/Oncology, Department of Medicine; Department of Anatomy and Cell Biology, State University of New York Downstate Medical Center, Brooklyn, NY, USA; 2 Department of Molecular Biology and Genetics, Bilkent University, Ankara, Turkey

## Abstract

**Background:**

In multiple myeloma (MM), increased neoangiogenesis contributes to tumor growth and disease progression. Increased levels of endothelial progenitor cells (EPCs) contribute to neoangiogenesis in MM, and, importantly, covary with disease activity and response to treatment. In order to understand the mechanisms responsible for increased EPC levels and neoangiogenic function in MM, we investigated whether these cells were clonal by determining X-chromosome inactivation (XCI) patterns in female patients by a human androgen receptor assay (HUMARA). In addition, EPCs and bone marrow cells were studied for the presence of clonotypic immunoglobulin heavy-chain (IGH) gene rearrangement, which indicates clonality in B cells; thus, its presence in EPCs would indicate a close genetic link between tumor cells in MM and endothelial cells that provide tumor neovascularization.

**Methods:**

A total of twenty-three consecutive patients who had not received chemotherapy were studied. Screening in 18 patients found that 11 displayed allelic *AR *in peripheral blood mononuclear cells, and these patients were further studied for XCI patterns in EPCs and hair root cells by HUMARA. In 2 patients whose EPCs were clonal by HUMARA, and in an additional 5 new patients, EPCs were studied for IGH gene rearrangement using PCR with family-specific primers for IGH variable genes (V_H_).

**Results:**

In 11 patients, analysis of EPCs by HUMARA revealed significant skewing (≥ 77% expression of a single allele) in 64% (n = 7). In 4 of these patients, XCI skewing was extreme (≥ 90% expression of a single allele). In contrast, XCI in hair root cells was random. Furthermore, PCR amplification with V_H _primers resulted in amplification of the same product in EPCs and bone marrow cells in 71% (n = 5) of 7 patients, while no IGH rearrangement was found in EPCs from healthy controls. In addition, in patients with XCI skewing in EPCs, advanced age was associated with poorer clinical status, unlike patients whose EPCs had random XCI.

**Conclusion:**

Our results suggest that EPCs in at least a substantial subpopulation of MM patients are related to the neoplastic clone and that this is an important mechanism for upregulation of tumor neovascularization in MM.

## Background

Increased neoangiogenesis has a governing role in the pathogenesis and progression of multiple myeloma (MM) [1], and is a reliable treatment target that has significantly improved outcome [2-6]. Increased circulating endothelial cells in cancer contribute to tumor neovascularization and growth [7,8].

We recently showed that in MM, elevated levels of circulating endothelial progenitor cells (EPCs) covary with disease activity, measured as serum M-protein and β_2_-microglobulin levels, underscoring the role of EPCs in MM pathogenesis [9]. Expression by EPCs of the early hematopoietic markers CD34 and CD133 indicates that these cells are derived from hematopoietic progenitor cells in the bone marrow (BM) [9-12]. Experimental evidence shows that in MM, EPCs both in circulation and in the BM express angioblastic features–a high capacity for vasculogenesis and a pro-angiogenic gene expression pattern–suggesting that these cells resemble embryonic angioblasts, and also supporting the thesis that BM EPCs help tumor neovascularization [8,11,13]. These observations prompted us to explore whether EPCs in MM display neoplastic features such as clonal restriction. Clonality in EPCs from MM patients was determined by X-chromosome inactivation (XCI) patterns using a highly polymorphic CAG repeat in the androgen receptor (*AR*) gene, a well-established method to assess clonality of tumor cells [14] based on random epigenetic inactivation of one of the two X chromosomes inherited from either parent [15]. Since the XCI pattern is stably inherited by all daughter cells, polyclonal cell populations reflect mosaicism, while in clonal neoplastic cells, the pattern is uniform [16]. We found that EPCs displayed significantly skewed XCI patterning (≥ 77% inactivation of one allele) in 64% of patients, while the hair root cells showed random XCI. Furthermore, there was evidence of immunoglobulin V_H _gene rearrangement in EPCs in a manner characteristic of clonal B lymphocytes, indicating that clonal EPCs are related to neoplastic MM cells at the genetic level. Thus, we suggest that myeloma-related genomic changes include the endothelial genome and constitute the basis for increased tumor neovascularization and growth.

## Methods

### Subjects

Twenty-three consecutive MM patients diagnosed by Southwest Oncology Group criteria [17] were studied according to protocols approved by the Institutional Review Board of State University of New York Downstate Medical Center, and informed consent was obtained in accordance with the Declaration of Helsinki. Clinical stage was determined according to Durie and Salmon [18]; in addition to stage, serum β_2_-microglobulin and albumin and, when feasible, tumor cell karyotype (Wycoff Heights Medical Center, Brooklyn, NY) constituted clinical prognostic indicators [19] (Table 1). Fifteen healthy hospital staff served as controls.

### EPCs

**Figure 1:**
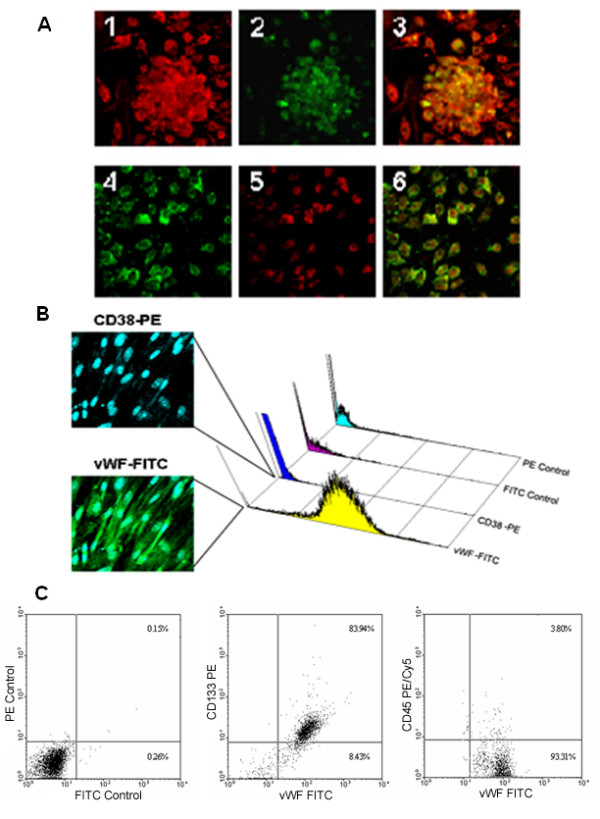
**Characterization of confluent EPC cultures expanded from BM expressing KDR, VE-cadherin, CD133, and vWF**. (A) Colony-forming and outgrowing endothelial cells from a representative patient are shown. A colony-forming unit expressing: (1) KDR (red), (2) VE-cadherin (green), and (3) merge shows independent cellular distribution of KDR and VE-cadherin. Outgrowing EPCs expressing: (4) KDR (green), (5) early hematopoietic antigen CD133 (red), and (6) merge show independent cellular localization of KDR and CD133. Cultures were maintained on 96-well, laminin-coated plates, where colony growth and confluent EPCs were observed after 14 and 18 days of culture, respectively. Indirect immunofluorescence was done with indicated antibodies, and appropriate isotype-matched serum with each of the primary antibodies was used as negative control (not shown). Images of the stained cells were digitally recorded on a confocal laser scanning microscope (Bio-Rad MRC 1024ES; Bio-Rad, Hercules, CA) and were generated at the projections of the z-stacks at 1024 × 1024 pixels. (B) Co-staining of EPCs grown from BM for expression of vWF (green), CD38 (red), and nuclear counterstain TO-PRO-3 (blue). Data from a representative patient show that EPCs are vWF-positive. Boxes on the left indicate patterns of immunocytochemical staining of the same EPCs; 3-D histogram on the right shows 2-color FACS analysis using anti-vWF-FITC, anti-CD38-PE, and isotype-specific control antibodies. (C) Two-color dot plot of primary EPC culture from a representative patient using anti-vWF-FITC, anti-CD133-PE, and anti-CD45-PE/Cy5 antibodies. Quadrants were set based upon isotype-specific controls for FITC and PE (and PE/Cy5, not shown). Numbers shown in the quadrants reflect percent of EPCs from which small, agranular debris was gated out based on forward and side scatter plots.

Confluent EPCs were outgrown from peripheral blood mononuclear cells (PBMCs) and from BM for DNA extraction. EPCs outgrown from PBMCs were preferentially used; however, when DNA from EPCs derived from PBMCs was not sufficient for the outlined studies, DNA from EPCs grown from BM was used. The EPC DNA for each patient was obtained from only one source, i.e., either from peripheral blood or from BM aspirate, as indicated in Tables 1, 2, 3. To grow EPCs, heparinized peripheral blood (20 ml), or a single-cell suspension of bone marrow (10 ml) was separated by Ficoll-Hypaque (Sigma Chemicals, St. Louis, MO) density-gradient centrifugation within 6 hours of collection. The mononuclear cell layer was resuspended in EndoCult Basal Medium supplemented with 20% fetal bovine serum (StemCell Technologies, Vancouver, Canada) and antibiotics (100 U/ml penicillin G, 100 μg/ml streptomycin). Ficoll-separated mononuclear cells from peripheral blood or bone marrow (7 × 10^6^/well) were grown to confluence in laminin-coated, 6-well plates (Becton Dickinson Labware, Bedford, MA) as previously described [9,20]. Under these culture conditions, EPC colonies are observed at 14 to 18 days, and confluence is reached 18 to 28 days after plating peripheral blood or bone marrow cells. To determine endothelial cell phenotype and purity of outgrown cells, EPCs were also grown on laminin-coated, 96-well plates (Becton Dickinson Labware) and subjected to two-color immunostaining with combinations of antibodies directed against endothelial cell markers, including vascular endothelial growth factor receptor-2 (kinase insert domain-containing receptor/fetal liver kinase-1 [KDR]), CD133, CD144 (vascular endothelial cadherin [VE-cadherin]), and von Willebrand factor (vWF) as we previously described [9]. Anti-CD38-PE (BD PharMingen, San Diego, CA) was employed to detect MM cells; TO-PRO-3 (Molecular Probes, Eugene, OR) was used to counterstain the nuclei of EPCs (Figure 1). Appropriate isotype-matched serum with each of the antibodies was used as negative control. Images of the stained cells were digitally recorded in the sequential mode on a confocal laser scanning microscope (Bio-Rad MRC 1024ES, Bio-Rad, Hercules, CA). Percent positive cells were quantitated in a minimum of 3 fields (original × 40) per well. In addition, viable EPCs, after detachment from laminin (Cell Dissociation Buffer, Invitrogen, Carlsbad, CA), were stained using direct immunofluorescence with indicated combinations of anti-vWF-FITC (Serotec, Raleigh, NC), anti-CD38-PE (BD PharMingen), anti-CD133-PE (Miltenyi Biotec, Auburn, CA), and anti-CD45-PECy5 (eBioscience, San Diego, CA), as well as isotype-specific control antibodies, and analyzed with a FACSort flow cytometer (BD Biosciences) as previously described [9]. To assure absence of contaminating plasma cells, bone marrow aspirates were also stained for the plasma cell marker CD38 using anti-CD38-PE and analyzed by flow cytometry (see Additional file 1).

### Human AR assay

Human androgen receptor gene assay (HUMARA) was used to examine XCI status of EPCs and non-endothelial cells to evaluate the clonal status of each population. Heterozygosity at the *AR *locus is a prerequisite for XCI evaluation by HUMARA. Androgen receptor locus was therefore examined in 18 female MM patients by polymerase chain reaction (PCR) assay in DNA extracted from PBMCs, as described previously [21]. Definitive polymorphism in the human *AR *locus was found in 11 of the 18 patients studied (Table 1, Cases 1–11). In the remaining 7 patients, the *AR *locus did not display obvious heterozygosity (data not shown) and therefore these patients were not candidates for evaluation of EPC clonality using HUMARA. Within a monoclonal cell population in a woman, all cells have the same combination of active and inactive X chromosomes. The maternally and paternally inherited X chromosomes can be distinguished by polymorphisms involving the number of tandemly repeated CAG repeats within the *AR *gene. Using *AR*-specific PCR primers that flank the CAG repeats, two PCR products of different sizes can be amplified from a heterozygous patient's genomic DNA. The primers that flank the *AR *CAG repeat sequence also span an *Hpa*II restriction site (CCGG) that is methylated and thereby protected from digestion when present on an inactive X chromosome, but is unmethylated and thereby susceptible to digestion when present on an active X chromosome. Thus, when *Hpa*II-treated DNA is subjected to PCR with *AR*-specific primers, copies of the inactive allele are amplified, whereas copies of the active allele are cleaved by *Hpa*II and cannot yield a PCR product. As a result, a single product is obtained after amplification of DNA from a clonal cell population from a heterozygous female patient. DNA was extracted from confluent EPCs, intact hair roots, and PBMCs using the QIAamp DNA Mini Kit (QIAGEN, Valencia, CA). DNA samples were divided into two identical aliquots, one of which was incubated overnight at 37°C with the methylation-sensitive restriction enzyme *Hpa*II for the digestion of unmethylated (or active) alleles. A second restriction enzyme, *Rsa*I, which recognizes a four-base pair sequence not present in the amplified region of the *AR *locus, was also included in the reaction to facilitate the *Hpa*II digestion process. Male DNA with cytogenetically verified 46XY karyotype was used as control for complete digestion.

Both digested and undigested DNA samples were amplified using *AR*-specific primers 5'-GTC CAA GAC CTA CCG AGG AG-3' and 5'-CCA GGA CCA GGT AGC CTG TG-3'. Amplicons were labeled by including a radioactive nucleotide (α-[^33^P]-dCTP) (NEN, PerkinElmer Life Sciences, Boston, MA) in the PCR. PCR products were separated on 8% denaturing gels, followed by autoradiography. Densitometric analyses of the alleles, performed at least twice for each sample using MultiAnalyst version 1.1 software (Bio-Rad), included normalization of the ratios based upon the non-digested samples by dividing the allele ratio of the digested sample by the ratio of the non-digested sample from the same specimen.

### IGH gene rearrangement

One-step PCR as described by Deane and Norton [22] was used to determine V_H _gene rearrangement within confluent EPCs and MM-cell-infiltrated BM aspirate samples. In each experiment, genomic DNA extracted from unseparated BM aspirate containing 10%-95% MM cells (Table 3) was used to identify the clonotypic IG V_H _gene rearrangement within the neoplastic plasma cells. V_H _genes were amplified from genomic DNA extracted from freshly obtained bone marrow samples and primary EPCs using a genomic DNA purification kit (Gentra Systems, Minneapolis, MN). A set of 7 V_H _family-specific PCR primers was used to isolate the clonally expressed V_H_D_H_J_H_, as described previously [22]. The forward primers were from the first framework region of the most common V_H _family members, and the single reverse J_H _primer was common to the 3' end of all six J_H _genes. PCR products were run on a 1.0% agarose gel, where the V_H _gene rearrangement was identified as a single band of 350 bp. For cloning and sequencing, DNA was extracted from the resolved 350-bp bands using the QIAquick Gel Extraction Kit (QIAGEN). Dideoxy sequencing (GENEWIZ, North Brunswick, NJ) was performed using the same V_H _primer that was added to the original PCR reaction. Nucleotide sequences of amplified DNA were identified by BLAST and compared by FASTA.

To rule out false-positive results due to contaminating plasma cells, the sensitivity of the PCR system to detect IGH rearrangement in EPCs was evaluated by quantitating the minimum contamination with tumor DNA required for IGH gene rearrangement. Thus, DNA samples from human umbilical vein endothelial cells (HUVECs), which do not have IGH rearrangement, were spiked with decreasing percentages of DNA obtained from the MM cell line U266, which has a clonotypic IGH rearrangement. In these experimental conditions, 2% or more of contaminating plasma cells were necessary to give positive results for IGH gene rearrangement (data not shown). As flow cytometry showed positivity of 0.1 ± 0.01% (n = 3) in EPCs for the myeloma cell antigen CD38, IGH rearrangement observed in these cultures was concluded to be a tumor-specific feature of EPCs. In addition to DNA extracted from HUVECs, DNA from EPCs outgrown from a healthy control were studied for V_H _rearrangement as negative controls for these experiments.

### Quantitation of rearranged IGH locus in primary EPCs by real-time PCR

To further rule out that plasma cell contamination in EPCs could be responsible for IGH rearrangement found in primary cultures, quantitation of EPCs containing the IGH rearrangement was performed using SYBR Green I real-time PCR. In B cell malignancies, quantitative real-time PCR assays have been established that use tumor-specific IGH rearrangements as quantitative markers of tumor cells [23]. The amount of fluorescence produced by this real-time PCR reaction is proportional to the starting DNA target number during the early phases of amplification. DNA from primary EPCs from Case 15, which contained the clonotypic rearrangement found in BM cells, was PCR-amplified using V_H_4A forward and J_H _reverse primers [22], and cloned using a TA Cloning Kit (Invitrogen, Carlsbad, CA) following recommended procedures. Briefly, about 5 ng of fresh PCR product was ligated to 50 ng of pCR2.1 at equal molar concentrations overnight at 16°C. Competent DH5α *Escherichia coli *were transformed with 20% of the ligation reaction and plated on LB agar with 100 μg/mL of ampicillin. Recombinant plasmid containing the ligated PCR-amplified IGH gene product was recovered with a QIAGEN Miniprep DNA purification system (QIAGEN) and digested with EcoRI to confirm presence of insert. Sequencing of cloned plasmid DNA using the M13 reverse primer (GENEWIZ) also confirmed the inserted patient-specific IGH rearrangement (data not shown).

Plasmid DNA containing human apolipoprotein B100 (ApoB) [24] was obtained from Kezhi Dai for use as an internal standard to normalize genomic DNA levels of IGH rearrangement in EPCs and BM cells from the same patient, as described for detection of minimal residual disease in acute lymphoblastic leukemia [25,26]. Quantitative PCR was performed using the qPCR Core Kit for SYBR Green I (Eurogenetec, San Diego, CA). Reactions performed in duplicate for IGH quantitation contained 10 ng of either EPC or BM cell genomic DNA from Case 15, and 2 × qPCR master mix containing 8 μM of either IGH primers (V_H_4 forward 5'-TCGGAGACCCTGTCCCTCACCTGCA-3', patient allele-specific reverse 5'-CCAGAGATCGAAGAACCAGTCTTC-3') or ApoB primers (ApoB forward 5'-GCAAG CAGAA GCCAG AAGTG A-3', ApoB reverse 5'-CCATT TGGAG AAGCAGTTTG G -3'). Amplification conditions, using an ABI Prism 7000 sequence detector equipped with a 96-well thermal cycler (Applied Biosystems, Foster City, CA), were 10 minutes at 95°C (to activate the enzyme) and 40 cyclesof denaturation at 95°C for 15 seconds and annealing/extension at 60°C for 1 minute. Data were collected and analyzed with ABI Prism SDS software v1.0 (Applied Biosystems, Foster City, CA). To obtain a standard curve for quantifying patient BM and EPC DNA, IGH and ApoB plasmids were diluted in 4-fold increments and subjected to quantitative PCR in duplicate as previously described [25]. Standard curves plotting cycle threshold (C_T_) versus log number of copies were produced with correlation coefficients greater than or equal to 0.97. Copy numbers of ApoB and rearranged IGH loci in EPCs and BM were obtained from these standard curves. Percent of cells bearing the IGH rearrangement was calculated as 100% × (log copy numbers of IGH/log copy numbers of ApoB).

### Statistical analysis

Data are presented as means ± standard deviations. Student's t test, two-tailed, was used to compare indicated groups. Associations between variables were evaluated by Pearson's r. Significance was set at p ≤ .05.

## Results and discussion

Confluent primary EPCs outgrown from patients' PBMCs or BM were the source of genomic DNA used in our experiments. Phenotypic characterization of PBMC- or BM-derived EPCs by immunocytochemistry and flow cytometry showed that at Days 14–18 of culture, EPCs expressed KDR, VE-cadherin, and CD133 antigens (Figure 1A). Specifically, flow cytometric analysis showed that 94 ± 2.5% (n = 4) of EPCs expressed the endothelial antigen vWF, while less than 0.3 ± 0.01% (n = 3) of EPCs expressed the neoplastic plasma cell marker CD38. A significant majority of vWF-positive EPCs expressed the early endothelial antigen CD133 and were null for the leukocyte-common antigen, CD45 (Figure 1 A–C). Persistence of CD133 expression by late EPCs is unusual since this antigen is lost during EPC maturation, and studies are underway in our laboratory to further assess expression of CD133 at the mRNA and protein levels in MM EPCs.

These data indicate that, irrespective of the source, i.e., PBMCs or BM, the EPC culture conditions, which lack exogenously added growth factors or cytokines (other than fetal bovine serum), and from which nonadherent cells are removed every three days, do not allow propagation of MM cells, which are known to be non-adherent, or other hematopoietic cells. In fact, the overwhelming vWF positivity observed at Days 14–18 indicated that cell types other than EPCs were not expanded in this culture system, indicating that culturing cells on laminin (and not methylcellulose), and in the absence of exogenously added factors, probably contributed to the absence of hematopoietic or stromal cell growth.

**Figure 2:**
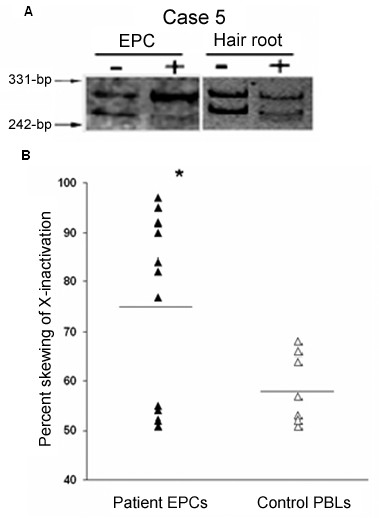
**X-chromosome inactivation patterns in EPCs from MM patients**. (A) X-chromosome inactivation status in a MM patient (Case 5, Table 1). XCI patterns were determined at least twice for each sample by incorporating α-[^33^P]-dCTP into the PCR reaction. For each sample, DNA was either undigested (-) or digested (+) with the methylation-sensitive restriction enzyme *Hpa*II. The PCR products were separated on 8% sequencing gels and subjected to autoradiography. Intensity of the specific bands corresponding to the *AR *gene demonstrates a skewed pattern of XCI (predominance of a single band) in EPCs, and a random pattern of XCI (presence of two bands of similar intensity) in hair root cells. A clonal population was defined as a cell population with greater than 77% expression of either one of the X-linked alleles. (B) Distribution of XCI patterns in MM patients and healthy controls. XCI patterns were determined in DNA from EPCs in MM patients (filled triangles), and PBMCs in healthy controls (open triangles) (*p = .05). For clarity, the y-axis begins at 40%.

To determine clonality within EPCs, we studied XCI patterns of these cells and compared them to XCI patterns of non-endothelial cells (hair root from the same patient) using HUMARA. Polymorphism in the human *AR *locus was found in 11 female patients, who were thus candidates for this analysis. As shown in Figure 2A, undigested DNA from EPCs or hair root cells yielded two alleles in a MM patient heterozygous for the analyzed polymorphism. In EPCs, digestion with the methylation-sensitive enzyme *Hpa*II targeted the unmethylated *AR *allele in all of the cells, resulting in a skewed XCI pattern, indicated by a single band, while in hair root cells, random XCI caused targeting and amplification of both *AR *alleles, as indicated by the two bands. Determination of XCI patterns in candidate patients showed that 7 of the 11 patients had skewing in XCI, indicated by a 77% or greater inactivation of one allele (Table 2). Of the 7 patients whose XCI patterns displayed skewing, 4 displayed extreme skewing (≥ 90% inactivation of one allele). Extremely skewed XCI is a rare event, reported in only 2–4% of 55–72-year-old women [27], and age was found not to contribute to skewing of XCI profiles in our patients (data not shown). It was important to establish that the X-inactivation profile of the EPCs in the study was not skewed due to a primary cause, which would be represented in the majority if not all of the tissues. Therefore, hair root, an ectoderm-derived tissue, was used in addition to mesodermal EPCs to examine XCI patterns. The difference in XCI patterns between EPCs and hair root cells in 6 patients (p = .03; Table 2) argues against a tissue-specific XCI distribution, as well as against primary causes for skewing, which would affect all cells [28]. Chemotherapy may cause secondary skewing in XCI; however, none of the patients were receiving chemotherapy. Preferential expansion of a single normal clone of EPCs is unlikely to be responsible for the skewing observed in XCI profiles, because DNA in each case was derived from numerous EPC-colony-forming units that expanded within the same time frame to contribute to the final confluent EPC population studied. To control for the skewing in XCI patterns observed in 2–5% of women in normal populations, we also compared patterns from patient EPCs to those from control PBMCs (Figure 2B), and found that mean skewing in XCI was significantly greater in patients compared to controls (p = .05), suggesting that the XCI pattern observed in MM patients' EPCs is unlikely to be part of a normal pattern. In the latter study, we ideally wanted to compare XCI patterns in EPCs between patients and controls. However, in the absence of exogenously added cytokines or growth factors, we were only able to obtain confluent EPCs in 1 of 7 healthy control patients. We therefore used PBMCs from normal controls to determine their XCI patterns.

To further explore genomic characteristics of MM EPCs, we determined V_H _gene rearrangement in tumor-containing BM cells and in confluent EPCs. Cells committed to the B cell lineage rearrange IG heavy- and light-chain genes at the DNA and mRNA levels, while non-B cells retain their genomic (non-rearranged) state. In MM, as in other late B cell malignancies, rearrangements in the IG heavy- and light-chain genes have been used to detect monoclonality [29,30]. We studied EPCs for evidence of IGH gene rearrangement by determining the V_H_DJ_H _region rearrangement. This approach was chosen because it allowed us to determine clonality in cells from both male and female patients; this approach also provided information about the clonal similarity between EPCs and neoplastic plasma cells, since all of the cells belonging to a clone would have the identical clonotypic V_H _region rearrangement.

**Figure 3:**
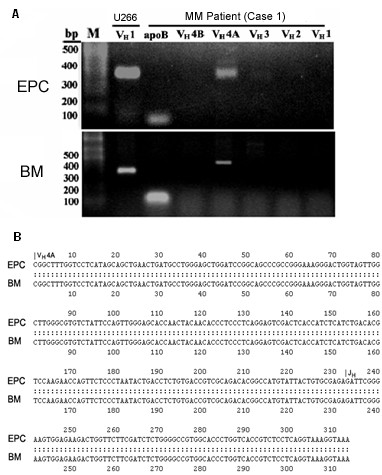
**PCR analysis and sequencing of immunoglobulin V_H _gene rearrangement in EPCs in MM patients**. A series of 7 V_H _family-specific primers (V_H_1–6) common to the most described members of the corresponding V_H _family, including two separate primers specific for the V_H_4 family (V_H_4A and -4B), were used as forward primers. A consensus J_H _primer was used as reverse primer to amplify rearranged DNA, producing an expected 350-bp PCR product as described by Deane and Norton [22]. (A) Agarose gel analysis of V_H_4A family-specific IGH gene rearrangement detected in EPCs and BM cells from a MM patient. Briefly, 1 μg of genomic DNA was amplified with each of the V_H _family primers and the constant J_H _primer, which were added at a concentration of 1 μM each. Products were run on 1% agarose gels and visualized with ethidium bromide staining. DNA from MM cell line U266, which contains a V_H_1-J_H _rearrangement, was amplified simultaneously as a positive control for rearrangement, shown as a 350-bp PCR product. Primers specific for apolipoprotein B served as a control to monitor the efficacy of PCR. M indicates marker (1 Kb Plus DNA Ladder [Invitrogen, Carlsbad, CA]). No products were detected from this patient's DNA with the V_H_5 or V_H_6 primers (not shown). (B) Sequence analysis of IGH rearrangement found in EPCs and BM cells from a MM patient. Sequences obtained from PCR amplification of IGH DNA in EPCs and BM cells shown from Case 14 were compared using FASTA. The 5' V_H_4A and J_H _regions identified by BLAST are shown above the sequences along with numbers indicating the length of the sequences analyzed. The V_H_4A-J_H _rearrangement in EPCs and BM cells from this patient has a 100% sequence overlap.

To study V_H _gene rearrangement in EPCs, we used a previously described panel of V_H _primers in conjunction with a single J_H _primer common to the 3' end of all six J_H _genes [22]. Each primer was specific to the first framework region of the most common V_H _family members [31]. As shown in Figure 3A, in which are presented results from a representative patient (Case 1), we found a single 350-bp V_H_4 product in EPCs, indicating that these cells had rearranged their IGH gene. In addition, BM plasma cells from the same patient also showed V_H _gene rearrangement, indicated by a similar 350-bp V_H_4 product. As summarized in Table 3, 5 of 7 patients (71%) showed the same single product after PCR amplification of EPC DNA as well as unseparated BM DNA, suggesting the presence of the same rearrangement in the V_H _gene both in EPCs and in BM cells. Two of these 5 patients also had extremely skewed XCI (Case 1), while the remaining 3 were not candidates for XCI analysis. The strong genetic linkage between EPCs and MM cells indicated by rearrangement of the V_H _gene in both cell types is strengthened by the 100% sequence similarity between the V_H _rearrangements and absence of V_H _mutations in EPCs and bone marrow cells (IMGT, the International ImMunoGeneTics information system, ). Furthermore, data from the same patient also showed that 51% of the patient's EPCs and 82% of the patient's BM cells contained the IGH rearrangement. This was accomplished using SYBR Green I quantitative real-time PCR, whereby copy numbers of IGH rearrangement were compared to those of apolipoprotein B100 (chromosome locus 2p24), which does not undergo rearrangement (see Additional file 2). This result is inconsistent with tumor contamination accounting for IGH rearrangement by PCR, since flow cytometric analysis did not show detectable numbers of plasma cells in EPC cultures. The results are also in accord with recently published data derived from 5 patients wherein an average of 18% of EPCs in MM patients were shown by fluorescent in situ hybridization to have the MM-specific chromosomal translocations [32].

In EPCs from 2 patients and 1 healthy control, there was no evidence of V_H _gene rearrangement. In addition, HUVECs also did not have V_H _gene rearrangement (Table 3). Thus, V_H _gene rearrangement in EPCs is not universal in MM, as supported by our finding of mosaic XCI patterns in 4 patients (36%) (Table 2). These findings are not unexpected, given the known genetic heterogeneity of MM [29]. Limitations of the primers in detecting rearrangements involving other V_H _gene regions, biallelic rearrangements that reduce the intensity of clonal amplifications, loss of primer annealing, or aberrant V_H _gene rearrangements within the myeloma clone may also have contributed to the lack of rearrangement in two patients [30,33]. At present, events downstream from V_H _gene rearrangement in EPCs, including IG transcription and translation, are being studied.

Our results are in agreement with previous publications in which a population of endothelial cells in chronic myeloid leukemia was shown to harbor the BCR/ABL fusion gene [34], and, more recently, identical non-random chromosomal translocations were found in endothelial cells and neoplastic B cells in non-Hodgkin's lymphoma [35], suggesting a genetic connection between these cells. Of the patients tested, karyotype analysis by G-banding did not reveal abnormalities that could be utilized to compare EPCs to MM cells (Table 1).

Taken together, these results show for the first time that in MM patients, EPCs, as distinct from non-hematopoietic cells, display skewing in XCI. Furthermore, EPCs have V_H _gene rearrangement identical to that found in neoplastic plasma cells, suggesting that there may be clonal identity between the two cell types. Genomic abnormalities in EPCs are a critical aspect of the increased angiogenesis associated with MM and the consequent detrimental effect on disease progression, and thus require further investigation.

Interesting clinical correlates of clonality were also present. In patients whose XCI studies showed skewing (n = 7), age was positively correlated with higher M protein levels (r = .80, p = .03), greater BM plasma cell infiltration (r = .97, p < .001), and more advanced stage of MM (r = .81, p = .03). In contrast, in patients in whom EPCs had random XCI (n = 4), these variables were unrelated or inversely related to age. The effect was particularly evident in the correlation of age with clinical stage (r = -.94, p = .06 for patients without skewing). Furthermore, as may be seen in Tables 1 and 2, a greater proportion of patients with EPCs that displayed random XCI had Stage 3 disease, compared to patients with XCI skewing in EPCs (3/4 vs. 1/7; P = .045, Fisher's Exact Test mid-P, two-tailed). The significance of these data is as yet unknown, especially since mean values of demographic and clinical characteristics did not differ between patients with and without XCI skewing in EPCs. However, these findings provide further evidence that the pathogenesis and clinical course of MM is differentially regulated based on genetic status of involved cell types. In this regard, it is perhaps relevant that constitutional XCI skewing is thought to mediate a susceptibility to breast cancer and ovarian cancer, and is hypothesized to be secondary to a skewed inactivation of an X-linked tumor suppressor gene, or perhaps to a low-penetrance susceptibility allele that is affected by the XCI patterns [36].

In our studies, hair root control DNA for XCI studies was not available for analysis in 3 patients who had skewed XCI in EPCs (Cases 3, 4, and 9, Table 2); thus, these patients were not included in the comparison of clonality between hair root and EPCs discussed for Table 2. We are planning to evaluate additional MM patients for constitutional XCI skewing and its possible role in predisposing patients to MM. This is especially relevant for our investigations of MM because we find this disorder to be prevalent in women in our inner-city patient population [37]. Prospective studies are now in progress examining clonality and severity of MM; these will further elucidate the role of EPC clonality in the natural history of this disease.

## Conclusion

These results underscore the necessity to explore the theses that MM cells and EPCs are derived from a multipotent progenitor [38] or a MM stem cell capable of self-renewal and differentiation [39], or are altered by factors that cause identical changes in MM cells and EPCs. Studies that examine the differentiation potential of single EPCs obtained from MM patients, and that compare EPCs to neoplastic plasma cells at the genetic level, are underway. Answering these questions will enhance our current understanding of MM angiogenesis and its relevance to the treatment of MM [40-42].

While this manuscript was being revised, Rigolin et al. [32] published data from 5 MM patients showing identical 13q14 deletion in circulating endothelial and bone marrow plasma cells.

## Competing interests

The author(s) declare that they have no competing interests.

## Authors' contributions

MB performed EPC cultures, IGH rearrangement studies, flow cytometry, and quantitative PCR, and significantly contributed to manuscript preparation. TÖ helped conceptualize the study. TÖ, SB, and CBA performed HUMARA experiments and helped with the manuscript. VV performed immunocytochemistry of EPCs. ELPS was instrumental in manuscript preparation, critical reading, and data analysis for this study. KD provided ApoB plasmid and primers and helped with quantitative PCR. OAB conceived the study, drafted the original manuscript and its submitted versions, and managed the patients. All authors read and approved the manuscript.

## Pre-publication history

The pre-publication history for this paper can be accessed here:



## Supplementary Material

Additional File 1 Flow cytometric analysis of bone marrow mononuclear cells from a representative MM patient showing CD38 positivity. Click here for file

Additional File 2 Quantitative real-time PCR analysis of IGH rearrangement in EPCs and BM cells from Case 15. Click here for file

## Figures and Tables

**Table 1:** Clinical characteristics of patients

**Case Number**	**Age/Sex**	**Source of EPC**	**%Plasma Cells in BM**^†^	**M protein (g/dL)**	**β_2_-microglobulin (mg/L)**	**Albumin (mg/mL)**	**Durie/Salmon Stage**	**Cytogenetics**^‡^
1	53/F	PBMC	10	1.2, IgG κ	1.6	2.3	2A	ND
2	62/F	PBMC	30	1.2, IgG λ	1.8	3.5	2A	Normal
3	60/F	PBMC	40	2.4, IgG κ	2.2	4.0	2A	ND
4	70/F	PBMC	80	6.0 IgG κ	3.0	3.5	3A	ND
5	54/F	PBMC	15	1.1, IgG κ	6.8	2.4	2A	ND
6	55/F	BM	10	3.0, IgG κ	2.0	3.4	2A	Normal
7	50/F	PBMC	30	4.8, IgG κ	4.6	3.7	3A^◆^	ND
8	46/F	PBMC	10	2.8, κ LC	4.9	3.9	3A	ND
9	52/F	BM	5	1.1, IgG κ	2.6	4.0	1A	ND
10	56/F	PBMC	30	2.0, λ LC	1.4	4.2	3A	ND
11	73/F	BM	10	3.0, IgG κ	2.4	3.8	1A	Normal
12	66/M	BM	10	3.0, IgG κ	2.0	4.1	1A	ND
13	56/F	BM	95	7.0, IgG κ	3.7	3.2	3A	Normal
14	54/F	BM	90	2.9, IgG λ	1.8	4.0	2A	ND
15	53/M	BM	95	3.6, IgA κ	3.0	3.0	3A	Normal
16	66/F	BM	60	5.4, IgA λ	3.5	1.9	3A	Normal

^†^Percent of plasma cells in BM indicates extent of myeloma involvement determined by histochemistry in the BM biopsies

^‡^Normal cytogenetics indicates results of chromosome banding studies and FISH performed using DNA probe D13S319 (Vysis Inc., Vysis, IL) for detection of 13q14.2 deletion

^◆^This patient had antecedent MGUS

"PBMC" peripheral blood mononuclear cells; "BM" bone marrow; "ND" Not done

**Table 2:** X-chromosome inactivation patterns in EPCs and hair root cells from MM patients. XCI patterns were determined in DNA from EPCs and hair root cells from 11 MM patients who had polymorphic AR status. The methylation status of a highly polymorphic CAG repeat in the AR gene was determined using the methylation-sensitive restriction enzyme *Hpa*II and PCR. Densitometric analyses of band intensities include normalization of the ratios, based upon the undigested samples. This was determined by dividing the allele ratio of the digested sample by the ratio of the undigested sample from the same specimen. This ratio corrects for preferential amplification of one allele, which often occurs for the shorter microsatellite allele. In EPCs from 6 patients whose EPCs and hair root cells were studied, the percent of skewing in XCI towards one inactivated allele was significantly greater in EPCs compared to hair root cells (*p = .03). "NA" indicates not available.

**Case Number**^†^	**EPC (Allele ratio)**	**Hair root cell (Allele ratio)**	**EPC Source**
1	95:5	50:50	PBMC
2	97:3	55:45	PBMC
3	92:8	NA	PBMC
4	90:10	NA	PBMC
5	82:18	54:46	PBMC
6	77:23	51:49	BM
7	55:45	55:45	PBMC
8	54:46	56:44	PBMC
9	84:16	NA	BM
10	51:49	Not done	PBMC
11	52:48	Not done	BM

Mean:	*75:25	53.5:46.5	

^†^Case number refers to cases detailed in Table 1

**Table 3:** IG V_H _gene rearrangement in EPCs and BM cells in MM. A single 350-bp band was amplified using the indicated V_H _primers in BM cells and confluent EPCs from each patient.

**Case Number**	**EPC (V_H _primer)**	**BM Cell (V_H _primer)**	**% Plasma cells in BM**	**EPC Source**
1	V_H_4A	V_H_4A	10	BM
2	V_H_4A	V_H_4A	30	BM
12	V_H_5	V_H_5	10	BM
13	V_H_4A	V_H_4A	95	BM
14	V_H_4A	V_H_4A	90	BM
15	No rearrangement	V_H_3	95	BM
16	No rearrangement	V_H_3	60	BM
				
Controls				
HUVECs	No rearrangement	---	---	---
Healthy subject	No rearrangement	---	---	---
