# Supplementary material for: Endothelial progenitor cells display clonal restriction in multiple myeloma
Source: BMC Cancer. 2006 Jun 22;6:161. doi: 10.1186/1471-2407-6-161 (PMC1557670; doi:10.1186/1471-2407-6-161)
Supplement: Additional File 1 — Flow cytometric analysis of bone marrow mononuclear cells from a representative MM patient showing CD38 positivity. [file 1471-2407-6-161-S1.doc]

1- Histogram showing bone marrow mononuclear cells from a representative MM patient with infiltration by CD38-PE-positive plasma cells (red histogram) and isotype-specific mouse IgG-PE control (empty histogram).
